# Lung Cancer Cell-Derived Exosomal let-7d-5p Down-Regulates OPRM1 to Promote Cancer-Induced Bone Pain

**DOI:** 10.3389/fcell.2021.666857

**Published:** 2021-05-26

**Authors:** Xihan Li, Yu Chen, Jialun Wang, Chengfei Jiang, Ying Huang

**Affiliations:** ^1^Department of Gastroenterology, Nanjing Drum Tower Hospital, The Affiliated Hospital of Nanjing University Medical School, Nanjing, China; ^2^Department of Pain Medicine, Nanjing Drum Tower Hospital, The Affiliated Hospital of Nanjing University Medical School, Nanjing, China

**Keywords:** exosome, miRNA, let-7d-5p, OPRM1, cancer-induced bone pain

## Abstract

Cancer-induced bone pain (CIBP) is the pain caused by metastasis of malignant tumors to the bone, accounting for more than half of all chronic cancer pain, which seriously affects the quality of life among tumor patients. Up to 40% of patients with advanced lung cancer suffer from CIBP. MicroRNA (miRNA) transfers between cells through exosomes, mediates cell-to-cell communication, and performs various biological functions. Studies have shown that miRNAs secreted by cancer can modify the tumor microenvironment, but whether exosome-mediated miRNA transfer plays a role in CIBP is still unknown. In this study, the expression levels of 15 miRNAs in exosomes derived A549 cells and 18 miRNAs in exosomes derived NCI-H1299 cells were significantly up-regulated, and qRT-PCR further confirmed that the level of let-7d-5p was increased most considerably. *In vitro*, exosomal let-7d-5p (EXO let-7d-5p) can be taken up by dorsal root ganglion (DRG) neurons and inhibit the protein level of the target gene opioid receptor mu 1 (OPRM1). EXO let-7d-5p was further confirmed to be involved in the generation and maintenance of CIBP *in vivo*. Our findings clarify the molecular mechanism of CIBP caused by the inhibition of OPRM1 by EXO let-7d-5p, providing new clues and intervention targets for the prevention and treatment of CIBP.

## Introduction

Lung cancer is one of the most aggressive tumors, and bone is the most common metastatic site of lung cancer (Lee et al., 2008; Wang et al., 2010; Jemal et al., 2011). Up to 40% of patients with advanced lung cancer have bone metastases, which are the primary source of pain and disability (Siegel et al., 2012). Patients with bone metastases have severe cancer-induced bone pain (CIBP), spinal cord compression, anemia, hypercalcemia or other nerve compression symptoms, which seriously affects the patients’ quality of life and reduces survival rate (Schneider et al., 2012; Ke et al., 2017). CIBP not only presents with persistent dull pain, but is often accompanied by unbearable breakthrough pain (Zhou et al., 2015). At present, there is a lack of effective treatment for CIBP. Although opioids have been widely used in CIBP, which cannot be fully controlled, and some patients cannot tolerate the dose-related side effects of opioids (respiratory depression, lethargy and constipation, etc.) (Christo and Mazloomdoost, 2008; Mantyh, 2013; Boland et al., 2015; Zhou et al., 2016). Therefore, it is urgent to study the potential molecular mechanism of CIBP and develop new therapeutic methods with good efficacy and low side effects.

Exosomes are vesicle-like bodies with a diameter of 30–120 nm that are actively secreted by cells (Singh et al., 2014; Hu et al., 2019). Exosomes are often cup-shaped when viewed under an electron microscope, but they are usually spherical in body fluids and can be released by different cells such as dendritic cells, epithelial cells, mast cells, fibroblasts and tumor cells (van Niel et al., 2006). Exosomes can be detected in peripheral blood, urine, saliva, cerebrospinal fluid, joint fluid and other parts, which play an important role in many physiological and pathological processes such as immune surveillance, inflammation and cancer development (Wahlgren et al., 2012). Tumor cells can actively secrete exosomes, which can carry lipids, proteins, mRNA, microRNA and DNA to participate in cell communication, cell migration, angiogenesis, tumor cell growth and drug resistance (Mathivanan et al., 2010; Kharaziha et al., 2012). Exosomes are carriers of miRNAs transfer between cells, which enable miRNA to avoid degradation in the transfer process and promote the effective uptake by target cells (He et al., 2014). MiRNA is a non-coding small RNA molecule regulating gene expression by targeting mRNA to inhibit its translation (Bartel, 2009). A variety of miRNAs have been detected in exosomes from different tumor cells, so the possible functions of miRNAs carried by exosomes in the tumor microenvironment have also been gradually concerned (Valadi et al., 2007; Taylor and Gercel-Taylor, 2008; Bartel, 2009; Mar-Aguilar et al., 2013). Studies have reported that changes in miRNA expression are related to the generation and improvement of pain, which initially revealed the role of miRNA expression in pain research. Zhang ZJ et al. found that miR-21 acted on Toll-like receptors (TLRs) to regulate the maintenance of neuropathic pain (Zhang et al., 2018). Pan ZQ believed that miR-23a regulated neuropathic pain by directly targeting CXCR4 via TXNIP/NLRP3 inflammasome axis in spinal glial cells (Pan et al., 2018). Fang BJ believed that miR-202 acted pivotal roles in the development of neuropathic pain partly through targeting RAP1A gene (Fang et al., 2019).

Our team injected exosomes from non-small cell lung cancer into nude mouse CIBP models and found that they developed mechanical hyperalgesia. However, we still know very little about how exosomes participate in tumor cells regulating bone metastasis and induce CIBP (Yuan et al., 2021). In this study, we revealed that EXO let-7d-5p secreted by non-small lung cancer cells could be delivered to spinal dorsal root neuron cells and inhibit the expression of OPRM1, thereby participating in the mechanism of CIBP production and maintenance. These data emphasize the role of exosomal miRNAs secreted by cancer cells in regulating CIBP, help to further clarify the regulatory mechanism of CIBP, and provide new clues and potential intervention targets for the prevention and treatment of CIBP.

## Materials and Methods

### Cell Lines

The human non-small cell lung cancer (NSCLC) cell lines A549 and NCI-H1299 were obtained from ATCC (Manassas, VA, United States). A549 cells were cultured in DMEM (GIBCO, United States) supplemented with 10% fetal calf serum (FCS) (GIBCO, United States), 100 U/ml penicillin and 100 U/ml streptomycins (GIBCO, United States). NCI-H1299 cells were cultured in RPMI1640 (GIBCO, United States) supplemented with 10% fetal calf serum (FCS) (GIBCO, United States), 100 U/ml penicillin and 100 U/ml streptomycin (GIBCO, United States). Cells were cultured at 37°C and 5% CO_2_.

### Exosomes Isolation

The A549 and NCI-H1299 cells were cultured in FBS-supplemented culture media to deplete exosomes, and the cell culture media were collected after 48-h cell cultures. Exosomes were separated by sequential ultracentrifugation, the cell culture media were centrifuged at 300 × *g* for 10 min to remove cells, then the supernatant was centrifuged at 2,000 × *g* for 20 min to collect exosomes. The supernatant was again centrifuged at 10,000 × *g* for 30 min, and the 0.22 μm filters were used to remove dead cells and cell debris. The collected exosomes were centrifuged at 120,000 × *g* for 90 min to obtain pelleted exosomes. The exosomes were washed and resuspended with PBS, then centrifuged again at 100,000 × *g* for 90 min, the pellets were resuspended in 1 ml cold PBS and stored at −80°C.

### Electron Microscopy

Exosomes were fixed with 2% paraformaldehyde and placed on a Formvar-carbon-coated electron microscope grid. As a control, the grids were negatively stained and embedded with 1%(w/v) uranyl acetate, and incubated at 4°C for 10 min. The excess fluid was removed, the mesh was sucked dry by Whatman filter paper, and imaged in JEM-200CX electron microscope (JEOL Ltd., Tokyo, Japan). The Total protein contents of exosomes were measured with the BCA protein detection kit (Abcam). The size distribution of exosomes was tracked by Nanoparticle tracking analysis (NTA).

### Western Blotting

Western blot was used to detect the presence of exosome-specific protein markers on the isolated vesicles. Total proteins were separated by 10%SDS polyacrylamide gel electrophoresis and transferred to nitrocellulose membranes (Millipore, Waltham, MA, United States). The membrane was washed with Tris-buffered saline (TBS) and incubated with 5% non-fat milk in TBST (TBS, 0.1% Tween 20) to block for 2 h. The membrane was then incubated with the primary antibody CD63 and CD9 overnight at 4°C, then incubated with the secondary antibody at room temperature for 1 h. The luminescence was observed using a chemiluminescence kit (Promega, Fitchburg, WI, United States).

### Primary Culture of DRG Neurons

Take BALB/c mice, dorsal root ganglion (DRG) were collected and put in DMEM medium supplemented with 1ml pancreatin and 0.1% collagenase type IV, digested at 150RPM for 40 min. The cell suspension was centrifuged at 500 RPM for 4 min, then the cells were resuspended in DMEM medium containing 10% fetal bovine serum and cultured at 37°C for 24 h.

### Exosome Uptake Analysis

Exosomes were fluorescently labeled with PKH67 membrane dye (Sigma-Aldrich, St. Louis, MO, United States). The Labeled exosomes were washed with 10ml PBS, collected by 100,000 × *g* ultracentrifugation, then resuspended in PBS. 50 μg/ml exosomes were incubated with DRG neuron cells for 24 h at 37°C, then fixed and stained with DAPI. The exosome uptake was observed by an LSCM (laser scanning confocal microscope; Zeiss LSM 710).

### RNA Extraction and Real-Time PCR

Total RNAs from exosomes or cells were extracted using the miRNeasy Mini Kit (Qiagen, Shanghai, China) according to the manufacturer’s protocol. The RNA quality was quantified using a NanoDrop 2000 spectrophotometer (Thermo Fisher Scientific). The expression profiles of miRNAs were analyzed by TaqMan low density array (TLDA). The relative expression levels of miRNAs were evaluated by the 2−ΔΔCt method using U6 snRNA as an internal reference. Quantitative RT-PCR (qRT-PCR) analysis was performed using the LightCycler 96-well block PCR system (Roche, Mannheim, Germany). The reverse transcription reactions were completed using TaqMan miRNA Reverse Transcription Kit and stem-loop primers for miRNAs.

### Prediction of miRNA Targets

With the help of bioinformatics analysis, miRNAs target genes prediction databases (TargetScan, miRWalk, miRDB and miranda) were used to predict the target genes of candidate miRNAs. The potential downstream target genes of let-7d-5p were predicted based on the intersections of four databases.

### Luciferase Reporter Assay

Human OPRM1 3 ’UTR fragment was amplified by PCR using human genomic DNA as a template. PCR products were cloned to the *Spe*I and *Hin*dIII sites in the pMIR-reporter plasmid polyclonal region and sequenced to confirm successful insertion. Firefly luciferase reporter gene system verified whether 3′-UTR of OPRM1 mRNA was targeted by let-7d-5p. Twenty-four hours after transfection, cell analysis was performed using a luciferase detection kit. All experiments were repeated three times.

### Animal Models

BALB/c mice (male, 20–25 g, 6–8 weeks old) were purchased from BK laboratory animals co., LTD., Shanghai, China, and raised under specific pathogen free (SPF) conditions with temperature control, including 12 h of the light-dark cycle. Forty-two BALB/c mice were used in experiments (*n* = 6/group). Mice can get food and water without restriction. The animals acclimated to the facility for a week before the study began. The overall health of the mice was checked regularly and their weights were measured every other day. All animal experiments were conducted by the animal experiment procedures approved by the Nanjing University Experimental Animal Ethics Committee. Animal handling follows the regulations of the Institutional Animal Care and Use Committee (IACUC) of Nanjing University. The tumor cells were injected as described earlier. In the CIBP model, male mice were anesthetized with isoflurane gas (2% isoflurane mixed with 100% oxygen). A distal femoral condyle arthrotomy was performed, and the patellar ligament was cut with scissors to expose the distal femoral condyle. 20 μL of cell suspension containing 10^6^ A549 tumor cells was injected into the intramedullary space of the femur. To prevent the cells from leaking out of the bone, the injection site was sealed with dental amalgam.

### Behavioral Analysis

Pain-related behavioral tests were performed on mice before and after tumor implantation. We used spontaneous flinches to assess persistent pain, and paw withdrawal latency (PWT) to assess mechanical pain. Before each experiment, the animals were placed in an experimental environment for 30 min to acclimatize. The number of spontaneous flinches was recorded during the 2-min observation period. Limb use was rated from 0 to 4 as follows: 4, normal limb use; 3, insignificant limping; 2, significant limping; 1, significant limping and lack of use of limbs; 0, complete lack of use of limbs. For PWT, the hind paws were stimulated with the von Frey single fiber. The mice were placed in a transparent cage with a metal mesh, and von Frey single fibers were used to stimulate the hind paws in ascending order. Each mouse was tested 5 times, the minimum stimulus intensity and the lowest von Frey fiber for inducing three or more positive reactions were considered to be the PWT. All the tests were conducted by subjects who were unaware of the experiment. The same assessment was repeated three times for all mice.

### Intrathecal Injection

As mentioned earlier, miRNA mimics (agomir-let-7d-5p) or inhibitors (antagomir-let-7d-5p) were administered intrathecally by implanting an intrathecal catheter. The PE10 catheter was inserted into the large pool for intrathecal implantation. 5 μl exosomes (1 mg/ml) or 5 μl miRNAs (20 μmol/L) were injected into the intrathecal catheter using a microscope syringe. Starting from the 15th day after tumor implantation, injections were given every 24 h for 7 consecutive days. After injection, flush the tube with 5 μl normal saline after injections. At the end of each experiment, laminectomy was performed to evaluate the catheterization, indicating that the catheterizations were correct.

### Immunohistochemistry

As described earlier, DRGs and spinal cord samples of mice were immunohistochemically analyzed. DRGs were continuously cut into 6 μm thick slices by a frozen microtome and mounted on glued slides. Specimens were placed in 3% BSA PBS solution, and incubated at room temperature for 1 h. Anti-OPRM1 was diluted at 1:100 for OPRM1 antibody staining. After incubation at room temperature for 3 h, slides were washed 3 times. The goat anti-rabbit IgG combined Alexa Fluor 555 were added, incubated at room temperature for 2 h, and the stationary slides were washed. Fluorescence images were obtained by laser scanning confocal microscopy (LSCM) using a Zeiss LSM 710 fluorescence microscope (Carl Zeiss Microscopy GmbH, Jena, Germany).

### Statistical Analysis

All the analyses were performed using GraphPad Prism software. The data were expressed as mean ± standard error of mean. One-way ANOVA was used to compare the data of each group. *P* < 0.05 was considered statistically significant. All *in vitro* data were analyzed from at least three separate experiments.

## Results

### Exosomes Characterization and Internalization in DRG Neuron Cells

**FIGURE 1:**
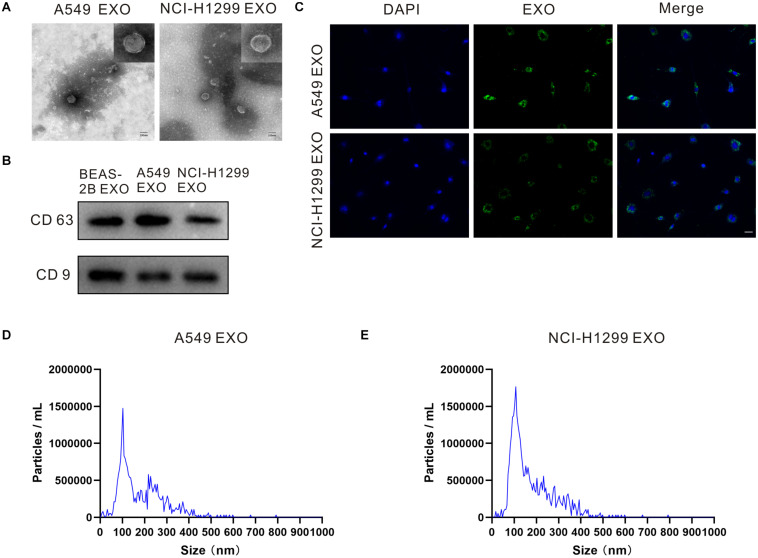
Characterizations of exosome morphology and specificity. **(A)** Representative electron micrographs of exosomes isolated from A549 and NCI-H1299 cells conditioned medium revealing the typical morphology and size. Scale bar represents 100 nm. **(B)** Western blot analysis showing abundant CD63 and CD9 in exosomes derived from the medium of A549 and NCI-H1299 cells. **(C)** The uptake of DRG cells after adding PKH67-labeled exosomes derived from A549 and NCI-H1299 cells. Images were taken 24 h after exosome addition by confocal microscope. Scale bar represents 50 μm. **(D,E)** Size distributions of A549 and NCI-H1299 exosomes were quantified using nanoparticle tracking analysis (NTA). All experiments were performed three times.

Exosomes can be actively released from a variety of cells including cancer cells. We incubated NSCLC cell lines A549 and NCI-H1299, normal human lung epithelial cell lines BEAS-2B in the exosome-free medium. Exosomes in the culture medium were separated by ultracentrifugation, then exosome pellets were resuspended in PBS, and the morphology of exosomes was examined by transmission electron microscopy (TEM). Figure1A and Supplementary Figure 1 showed that exosomes isolated from A549, NCI-H1299 and BEAS-2B cells had uniform cup shapes and vesicle sizes of about 100 nm. Besides, western blot results showed that these microcapsules were positive for exosome markers CD63 and CD9 (Figure 1B). In order to test the internalization of exosomes in DRG neuron cells, exosomes isolated from A549 and NCI-H1299 cells were labeled with PKH67 dye (green), washed thoroughly, and then added to DRG neuron cells. The uptake of exosomes from the recipient cells was observed under a confocal microscope. Almost all DRG neuron cells showed green signals (Figure1C). These results indicated that A549 and NCI-H1299 cell exosomes were internalized by DRG neuron cells. NTA revealed that A549 exosomes are 101.4 ± 42.4 nm in diameter and NCI-H1299 exosomes are 104.7 ± 39.1 nm in diameter (Figures 1D,E). BCA protein assay revealed that the concentration of proteins was 240 μg/ml in A549 exosomes and 280 μg/ml in NCI-H1299 exosomes.

### Identifying Exosomal miRNAs Markedly Secreted by Lung Cancer Cell Lines

**FIGURE 2:**
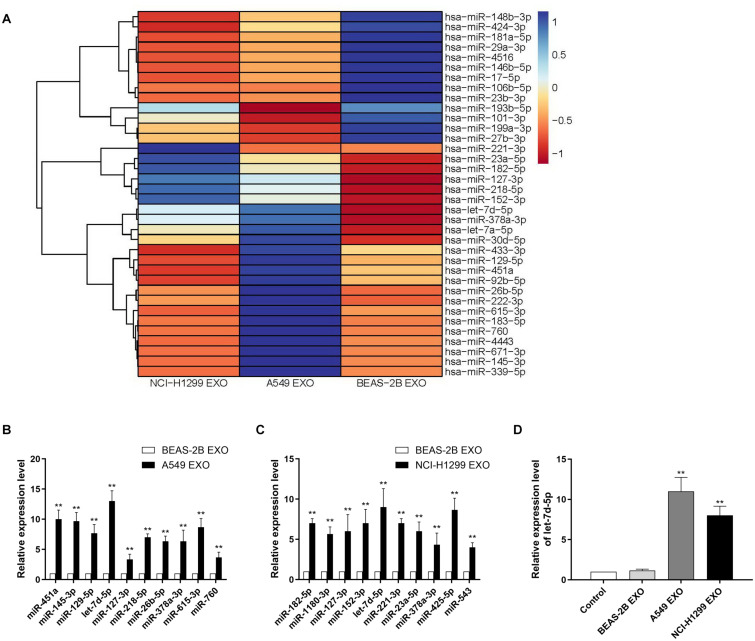
Identifying exosomal microRNAs secreted by NSCLC lines. **(A)** The expression profile of miRNAs in exosomes secreted by NSCLC lines. Exosomes from non-cancerous cells (BEAS-2B) were used as a normalization control. Blue color denotes higher expression, and red color denotes lower expression relative to the control. **(B)** qRT–PCR analysis revealing the expression of miRNAs in A549 cell–derived exosomes. **(C)** qRT-PCR analysis revealing the expression of miRNAs in NCI-H1299 cell–derived exosomes. **(D)** The expression of let-7d-5p in A549 and NCI-H1299 groups was up-regulated after treated DRG neuron cells with exosomes extracted from A549, NCI-H1299 and BEAS-2B cells detected by qRT-PCR. **p < 0.01. All experiments were performed three times.

Exosomes contain multiple coding and non-coding RNAs. Total RNAs from A549, NCI-H1299 and BEAS-2B cell exosomes were extracted and then analyzed using TLDA for exosomal miRNAs. We compared human NSCLC A549, NCI-H1299 and normal lung epithelial cells BEAS-2B, then quantitatively analyzed up to 768 miRNAs (Figure 2A). Comprehensive microarray analysis showed that compared with BEAS-2B cell exosomes, there were 24 miRNAs with a fold difference of more than 2 folds in A549 cell exosomes, of which 15 were up-regulated and 9 were down-regulated (Supplementary Table 1). We selected 10 miRNAs for qRT-PCR verification, and the results showed that the variation trend was consistent with the microarray results (Figure 2B). H1299 and BEAS-2B cell exosome miRNAs were also quite different. There were 29 miRNAs with a fold difference of more than 2 folds, of which 18 were up-regulated and 11 were down-regulated (Supplementary Table 2). We selected 10 miRNAs for qRT-PCR verification, the results also showed that the variation trend was consistent with the microarray results (Figure 2C). We found that the contents of let-7d-5p in A549 and NCI-H1299 cell exosomes were higher than other miRNAs. Compared with the control BEAS-2B cell exosomes, let-7d-5p was were up-regulated 13 times in A549 cell exosomes and 9 times in NCI-H1299 cell exosomes. We incubated DRG neuron cells with exosomes extracted from A549, NCI-H1299 and BEAS-2B cells, and qRT-PCR detection also confirmed that let-7d-5p expression was up-regulated in A549 and NCI-H1299 groups (Figure 2D).

### OPRM1 Is the Functional Target of let-7d-5p in DRG Neuron Cells

**FIGURE 3:**
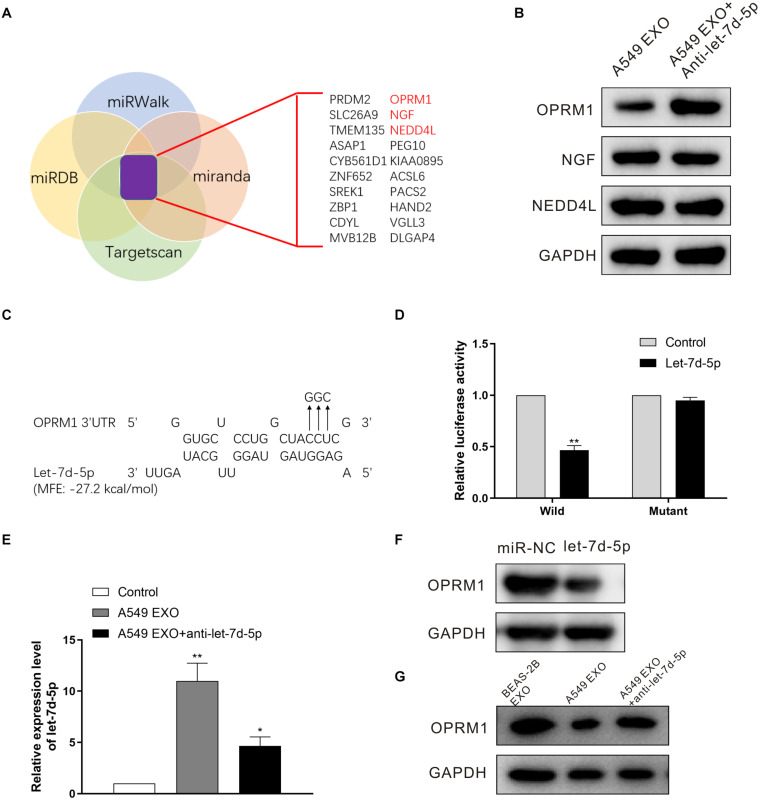
OPRM1 is the functional target of let-7d-5p in DRG neuron cells. **(A)** A diagram illustrating of target genes analysis. **(B)** Western blotting analysis of OPRM1, NGF and NEDD4L in DRG neuron cells with A549 exosomes plus anti-let-7d-5p lentivirus. GAPDH was used as loading control. **(C)** Predicted consequential binding sites of let-7d-5p and OPRM1 3′UTR. **(D)** Let-7d-5p suppressed the luciferase activity of the luciferase reporters carrying OPRM1 3′-UTR. Both wild-type (UTR-WT) or mutant (UTR-mut) reporters were introduced into 293T cells by transfection and then incubated with the let-7d-5p mimics. **P < 0.01. **(E)** The expression of let-7d-5p in A549 exosome plus anti-let-7d-5p lentivirus groups was down-regulated detected by qRT-PCR. *P < 0.05, **P < 0.01. **(F)** The relative expression levels of OPRM1 in DRG neuron cells infected with miR-NC and let-7d-5p lentivirus detected by Western blot. **(G)** The relative expression levels of OPRM1 in DRG neuron cells treated with BEAS-2B exosomes, A549 exosomes and A549 exosomes plus anti-let-7d-5p lentivirus detected by Western blot. All experiments were performed three times.

In order to confirm the targets of let-7d-5p, we used four target prediction databases (Targetscan, miRWalk, miRDB and miranda) for computer analysis. The results showed that pain-related receptors OPRM1, NGF (nerve growth factor) and NEDD4L (neural precursor cell-expressed developmentally down-regulated gene 4-like) were overlapped in all databases (Figure 3A). We treated the DRG neuron cells with A549 exosomes before infected with anti-let-7d-5p lentivirus, then detected the expression of OPRM1, NGF and NEDD4L in DRG neuron cells by Western blot. The results showed that there was no significant difference in the expression of NGF and NEDD4L. In contrast, the expression of OPRM1 was significantly up-regulated (Figure 3B), suggesting that the expression of OPRM1 may be regulated by let-7d-5p. Further computer analysis showed that the “UACCUC” site in the 3 ’UTR region of OPRM1 was complementary to the conserved sequence “GAGGUA” of let-7d-5p (Figure 3C). We cloned the 3’UTR binding site of OPRM1 into the luciferase gene and performed luciferase reporter gene analysis. The results confirmed that let-7d-5p can directly complement the 3′UTR of the OPRM1, let-7d-5p inhibited the 3′UTR luciferase activity of OPRM1, while let-7d-5p binding site mutations eliminated these inhibitory effects (Figure 3D). We treated DRG neuron cells with exosomes extracted from A549, NCI-H1299 and BEAS-2B cells, and qRT-PCR confirmed that the expression of let-7d-5p was up-regulated in A549 and NCI-H1299 groups. We further infected DRG neuron cells with anti-let-7d-5p lentivirus, qRT-PCR confirmed that the expression of let-7d-5p was down-regulated (Figure 3E). Western blot results showed that the overexpression of let-7d-5p significantly reduced the expression of OPRM1 in DRG neuron cells in let-7d-5p lentivirus (Figure 3F) and A549 exosome groups, however, the expression of OPRM1 was not significantly inhibited in BEAS-2B exosome and A549 exosome plus anti-let-7d-5p lentivirus groups (Figure 3G).

### Effects of Exosomes From Lung Cancer Cell Lines on CIBP–Related Behaviors

**FIGURE 4:**
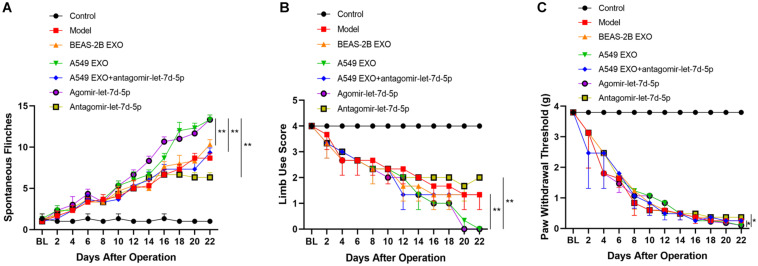
Effect of lung cancer cell lines-derived exosomes on pain-related behaviors in CIBP mice. Spontaneous flinches **(A)**, limb-use score **(B)** and paw withdrawal threshold **(C)** were evaluated before preoperative or 2, 4, 6, 8, 10, 12, 14, 16, 18, 20, 22 days after femoral inoculation. A549 exosomes and agomir-let-7d-5p significantly increased the number of flinches, reduced scores of limb use and paw withdrawal threshold. Data are presented as means ± SEM (*n* = 6/group). *P < 0.05, **P < 0.01. All experiments were performed three times.

As described earlier, the pain behavior of CIBP mice was evaluated before and after femoral inoculation. On the 15th day after femoral inoculation (Supplementary Figure 2), CIBP mice were given the intrathecal injection of exosomes and miRNAs every 24 h for 7 consecutive days. The results showed that intrathecal injection of A549 exosomes and agomir-let-7d-5p enhanced the pain behavior of CIBP mice, while antagomir-let-7d-5p inhibited the pain enhancement caused by A549 exosomes. Also, intrathecal injection of antagomir-let-7d-5p alone alleviated the pain of CIBP mice. CIBP mice in A549 exosome and agomir-let-7d-5p groups (*n* = 6) showed an increased number of spontaneous flinches (*P* < 0.01). Compared with the A549 exosome group, combined injection of antagomir-let-7d-5p inhibited the increase of spontaneous flinches caused by A549 exosomes, while intrathecal injection of antagomir-let-7d-5p alone reduced the spontaneous flinches of CIBP mice (Figure 4A). In terms of limb use scores, mice in the A549 exosome and agomir-let-7d-5p groups showed lower limb use scores and PWT than the BEAS-2B exosome group (*P* < 0.05). Compared with the A549 exosome group, combined injection of antagomir-let-7d-5p could improve the reduction of limb use scores and PWT caused by A549 exosomes, while intrathecal injection of antagomir-let-7d-5p alone enhanced the limb use scores and PWT in CIBP mice (Figures 4B,C).

**FIGURE 5:**
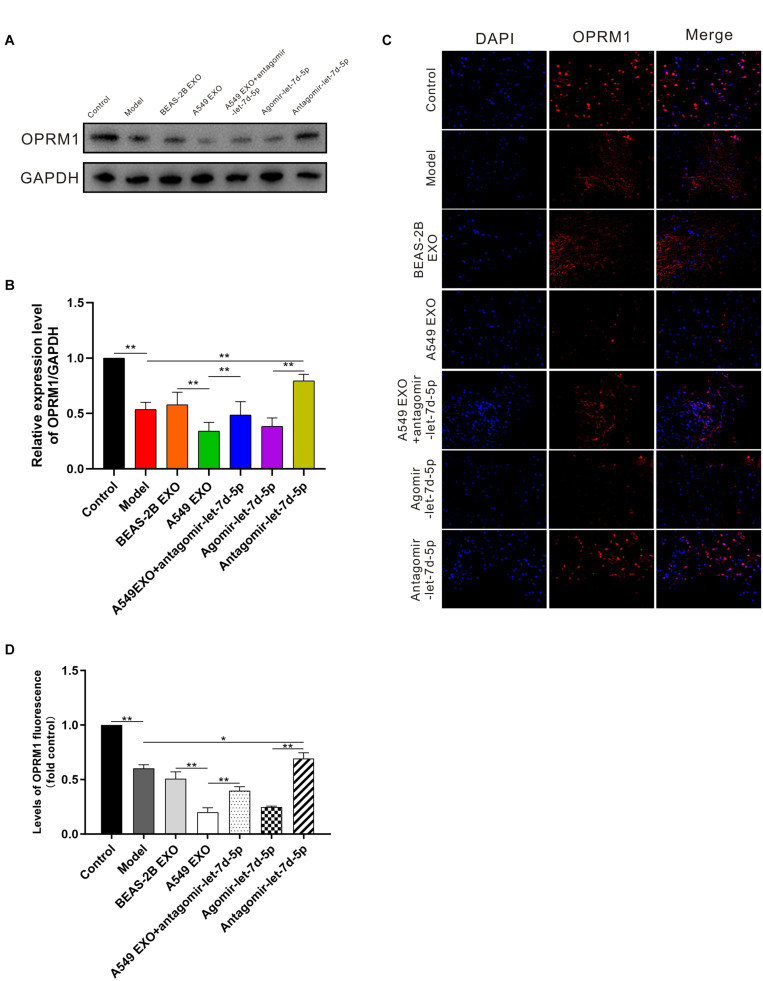
Expression of OPRM1 in the dorsal root ganglion (DRG) of CIBP mice. **(A,B)** One week after intrathecal injection, western blot analysis showed that in the DRG, OPRM1 protein was significantly reduced in A549 exosome and agomir-let-7d-5p groups. **(C,D)** Immunofluorescent staining showed that OPRM1 immunoreactivity was significantly reduced in the DRG in A549 exosome and agomir-let-7d-5p groups. Levels of OPRM1 fluorescence in tissues were quantified using Image J software. Scale bar = 50 μm. *P < 0.05, **P < 0.01. Datas are presented as mean ± SEM (*n* = 6/group). All experiments were performed three times.

We further studied the expression of OPRM1 in the DRG of mice. Seven days after intrathecal injection of exosomes and miRNAs, western blot analysis showed that the expression of OPRM1 protein in DRG in A549 exosome and agomir-let-7d-5p groups was significantly lower than that of the BEAS-2B exosome and model groups (*P* < 0.01). In contrast, the expression of OPRM1 protein in the A549 exosome combined antagomir-let-7d-5p group did not decrease significantly. Moreover, OPRM1 protein was slightly increased in antagomir-let-7d-5p group compared with that in the model group (Figures 5A,B). We further studied the immunofluorescence staining of OPRM1 in the DRG. In the DRG, OPRM1 immunoreactive fibers and neurons are mainly distributed in the superficial layer. The immunoreactivity of OPRM1 in A549 exosome and agomir-let-7d-5p groups was significantly reduced (*P* < 0.01), which was consistent with the quantitative results of western blot analysis. Compared with the A549 exosome group, the intrathecal combined delivery of antagomir-let-7d-5 increased the expression of OPRM1 in the DRG. Besides, OPRM1 was expressed at low levels in the DRG of CIBP mice, but antagomir-let-7d-5p increased the expression of OPRM1 (Figures 5C,D).

## Discussion

Bone metastasis can cause severe pain, pathological fractures and spinal cord compression, which can seriously affect the quality of life of cancer patients (Siegel et al., 2012). Although some clinical progress has been made, CIBP remains an important challenge for clinicians (Ke et al., 2017). There is an urgent need to further understand the underlying mechanism of CIBP, develop treatments based on specific mechanisms, and improve the patients’ quality of life. The mechanism of CIBP is complex, and may involve neuropathic pain and inflammatory pain (Schneider et al., 2012). In this study, non-small cell lung cancer cell line exosome miRNAs were screened, and the expression of exosome miRNAs and their functions in the occurrence and maintenance of tumor-mediated chronic pain were analyzed in the CIBP model, to provide new clues and potential intervention targets for the prevention and treatment of CIBP.

In this study, A549 exosomes and NCI-H1299 exosomes labeled with PKH67 were added to DRG neuron cells for co-incubation. PKH67 fluorescence was observed in DRG neuron cells, indicating that A549 exosomes and NCI-H1299 exosomes could be absorbed by DRG neuron cells. We further injected A549 cells directly into the femoral bone marrow cavity of nude mice to construct CIBP models. On the 15th day after inoculation, exosomes and miRNAs were injected intrathecally for seven consecutive days, and the results showed that mechanical hyperalgesia in the A549 exosome group was significantly earlier than that in the BEAS-2B exosome group. The above research results indicate that exosomes secreted by non-small cell lung cancer cells may be involved in the maintenance of CIBP. To further understand how exosomes play a role in the development of CIBP, we performed miRNA array analysis on A549 exosomes and NCI-H1299 exosomes. Compared with BEAS-2B exosomes, 15 up-regulated miRNAs and 9 down-regulated miRNAs were found in A549 exosomes (changes more than 2 folds), 18 up-regulated miRNAs and 11 down-regulated miRNAs were found in NCI-H1299 exosomes (changes more than 2 folds). In the subsequent RT-QPCR analysis, we confirmed that the most significantly up-regulated miRNA was let-7d-5p. DRG neuron cells were transfected with A549 exosomes and NCI-H1299 exosomes, and the expression of let-7d-5p was also up-regulated.

Let-7d-5p is associated with the development of breast cancer, ovarian cancer and other tumors, but its relationship with pain has not been reported (Hilly et al., 2016; Uhr et al., 2019; Zhang et al., 2019). We predicted and analyzed the possible targeted genes of let-7d-5p, including OPRM1, NGF and NEDD4L. NGF, a protein that induces nerve growth, was first discovered 60 years ago by Rita Levi-Montalcini (Zeliadt, 2013). Studies have found that NGF is also related to intractable pain (Hirose et al., 2016). NEDD4L is an effective Na_v_s post-translational regulator. Down-regulation of NEDD4L can lead to hyperexcitability of DRG neurons and participate in pathological pain (Laedermann et al., 2013). We infected DRG neuron cells with antagomir-let-7d-5p lentivirus, and western blot detected OPRM1, NGF and NEDD4L in DRG cells. It was found that there was no significant difference in the expression of NGF and NEDD4L, while OPRM1 was significantly up-regulated. Also, we added A549 exosomes and NCI-H1299 exosomes to DRG neuronal cells. The qRT-PCR results confirmed that let-7d-5p expression was up-regulated, and western blot confirmed that OPRM1 expression was significantly down-regulated. We further constructed a wild-type and mutant dual-luciferase reporter gene system at the binding site of let-7d-5p and OPRM1 3’UTR. The results also confirmed that let-7d-5p could directly bind to the OPRM1 3’UTR complementary and inhibit the expression of luciferase. The above results suggested that let-7d-5p in exosomes secreted by non-small cell lung cancer cells could regulate the expression of OPRM1 in DRG cells.

We further studied the changes in pain after intrathecal injection of exosomes and miRNAs in CIBP mice, as well as the changes in OPRM1 expression in the DRG. The results showed that intrathecal injection of A549 exosomes and let-7d-5p significantly enhanced the pain behavior of CIBP mice, while antagomir-let-7d-5p inhibited the pain enhancement caused by A549 exosomes. And the expression of OPRM1 in the DRG was consistent with the changes in hyperalgesia mediated by A549 exosomes and let-7d-5p. We demonstrated that exosomes from NSCLC and let-7d-5p were important pain-inducing factors of CIBP caused by NSCLC, which caused aggravation of pain in CIBP mice by inhibiting OPRM1 expression.

In conclusion, understanding the role and function of tumor-derived exosomes is important for understanding CIBP. We construct the human lung cancer CIBP model, in order to use these models to study the possible mechanism of human lung cancer CIBP. This study provides evidence that let-7d-5p transferred from exosomes derived from non-small cell lung cancer cells promotes the development of CIBP by targeting OPRM1, but further studies are needed both preclinically and clinically to develop a new potential therapy, which can alleviate CIBP effectively, and increase the functional status and quality of life of CIBP patients.

## Data Availability Statement

The original contributions presented in the study are included in the article/Supplementary Material, further inquiries can be directed to the corresponding author/s.

## Ethics Statement

The animal study was reviewed and approved by Nanjing University Experimental Animal Ethics Committee.

## Author Contributions

XL and YH designed the experiment, drafted the manuscript and revised it. XL, YC, and JW carried the experiment. CJ analyzed the data. All authors read and approved the final manuscript.

## Conflict of Interest

The authors declare that the research was conducted in the absence of any commercial or financial relationships that could be construed as a potential conflict of interest.

## Abbreviations

CIBP: Cancer-induced bone pain
miRNA: MicroRNA
NSCLC: non-small cell lung cancer
EXO: exosome
DRG: dorsal root ganglion
OPRM1: opioid receptor mu 1
TLR: Toll-like receptor
FCS: fetal calf serum
TBS: Tris-buffered saline
LSCM: laser scanning confocal microscope
TLDA: TaqMan low density array
SPF: specific pathogen free
IACUC: Institutional Animal Care and Use Committee
PWT: paw withdrawal latency
NGF: nerve growth factor
NEDD4L: neural precursor cell-expressed developmentally down-regulated gene 4-like.
