# Supplementary material for: Lung Cancer Cell-Derived Exosomal let-7d-5p Down-Regulates OPRM1 to Promote Cancer-Induced Bone Pain
Source: Front Cell Dev Biol. 2021 May 26;9:666857. doi: 10.3389/fcell.2021.666857 (PMC8188355; doi:10.3389/fcell.2021.666857)
Supplement: Supplementary file 1 [file Data_Sheet_1.DOCX]

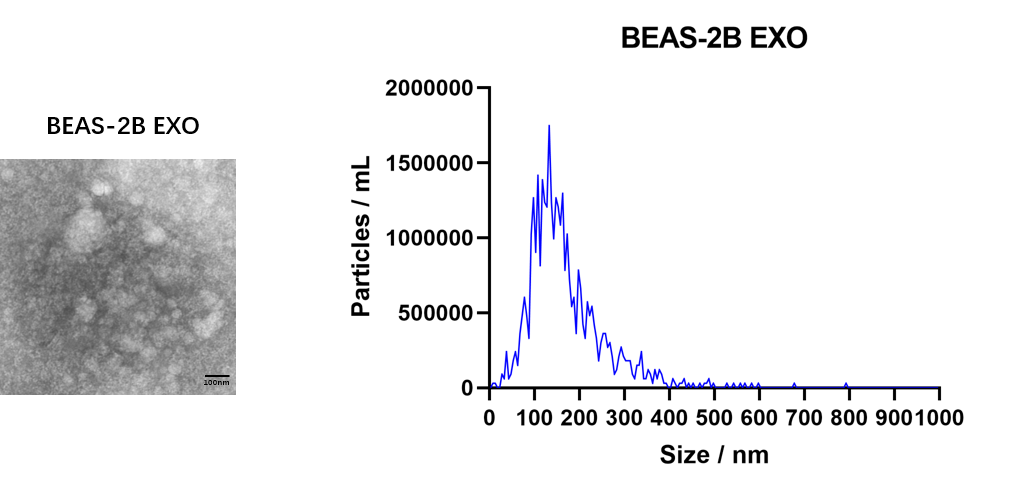


**Figure S1. Characterizations of exosome morphology and specificity**. A, Representative electron micrographs of exosomes isolated from BEAS-2B cells conditioned medium revealing the typical morphology and size. Scale bar represents 100 nm. B, Size distributions of BEAS-2B exosomes were quantified using nanoparticle tracking analysis (NTA).


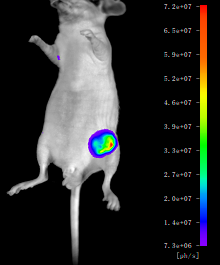


**Figure S2.** An examples of bioluminescent imaging data from CIBP mice 15 days post inoculation.

**Table S1.** Upregulated miRNAs in A549 exosomes compared with BEAS-2B exosomes determined by TaqMan Low Density Assay

| miRNA | ΔCt_A_ | ΔCt_B_ | ΔΔCt |
| --- | --- | --- | --- |
| miR-451a | 1.23 | 14.13 | -12.90 |
| miR-145-3p | 3.92 | 15.27 | -11.35 |
| miR-129-5p | 2.68 | 10.83 | -8.15 |
| let-7d-5p | 2.37 | 16.93 | -14.56 |
| miR-127-3p | 5.42 | 11.33 | -5.91 |
| miR-218-5p | 2.29 | 7.88 | -5.59 |
| miR-26b-5p | 5.19 | 12.51 | -7.32 |
| miR-378a-3p | 7.52 | 14.97 | -7.45 |
| miR-615-3p | 3.27 | 11.53 | -8.26 |
| miR-760 | 2.71 | 9.86 | -7.15 |
| miR-671-3p | 8.20 | 17.58 | -9.38 |
| miR-339-5p | 2.86 | 8.77 | -5.91 |
| miR-92b-5p | 5.17 | 11.40 | -3.23 |
| miR-30d-5p | 3.65 | 9.37 | -5.72 |
| miR-433-3p | 7.38 | 12.49 | -2.11 |

The different Ct value between two groups was calculated by ΔΔCt method: ΔCt_A_= Ct_target miRNA_ – Ct_U6_ _snRNA_; ΔCt_B_ = Ct_target miRNA_ − Ct_U6 snRNA_; ΔΔCt = ΔCt_A_– ΔCt_B_

**Table S2.** Upregulated miRNAs in NCI-H1299 exosomes compared with BEAS-2B exosomes determined by TaqMan Low Density Assay

| miRNA | ΔCt_N_ | ΔCt_B_ | ΔΔCt |
| --- | --- | --- | --- |
| miR-182-5p | 3.41 | 14.34 | -10.93 |
| miR-1180-3p | 4.17 | 14.91 | -10.74 |
| miR-127-3p | 4.35 | 13.57 | -9.22 |
| miR-152-3p | 2.74 | 13.47 | -10.73 |
| let-7d-5p | 2.45 | 14.73 | -12.28 |
| miR-221-3p | 7.03 | 15.22 | -8.19 |
| miR-23a-5p | 5.32 | 14.59 | -9.27 |
| miR-378a-3p | 6.18 | 13.73 | -7.55 |
| miR-425-5p | 7.97 | 16.73 | -8.76 |
| miR-543 | 5.73 | 13.46 | -7.73 |
| miR-485-3p | 8.68 | 15.16 | -6.48 |
| miR-129-5p | 9.85 | 15.14 | -5.29 |
| miR-145-3p | 3.33 | 9.69 | -6.36 |
| miR-28-3p | 4.25 | 9.49 | -4.24 |
| miR-3656 | 7.58 | 13.16 | -5.58 |
| miR-433-3p | 9.33 | 15.92 | -6.59 |
| miR-514a-3p | 4.96 | 10.29 | -3.33 |
| miR-671-3p | 3.12 | 8.35 | -2.23 |

The different Ct value between two groups was calculated by ΔΔCt method: ΔCt_N_= Ct_target miRNA_ – Ct_U6_ _snRNA_; ΔCt_B_ = Ct_target miRNA_ − Ct_U6 snRNA_; ΔΔCt = ΔCt_N_– ΔCt_B_
